# Accuracy of a subcutaneous continuous glucose management system in critically ill patients

**DOI:** 10.1186/2197-425X-3-S1-A291

**Published:** 2015-10-01

**Authors:** L Engelhardt, T Wollersheim, J Pachulla, R Mörgeli, F Balzer, K Mai, S Weber-Carstens

**Affiliations:** Campus Virchow & Campus Mitte, Anesthesiology and Operative Intensive Care Medicine, Charité Universitätsmedizin Berlin, Berlin, Germany; Department of Endocrinology, Diabetes and Nutrition, Charité Universitätsmedizin Berlin, Berlin, Germany; Berlin Institute of Health (BIH), Max Delbrück Center for Molecular Medicine and Charité Universitätsmedizin Berlin, Berlin, Germany

## Introduction

Continuous glucose management (CGM) has not yet been implemented to daily routine in the intensive care unit (ICU) setting. CGM systems aim to improve glycemic control, and consequently patient outcome.

## Objectives

The main purpose of this study was to evaluate accuracy of the subcutaneous Medtronic Sentrino® CGM system in critically ill patients.

## Methods

Inclusion criteria were an expected length of stay in the ICU of at least 72h, age ≥ 18 years, and availability of informed consent given by patient or legal proxy. Sensors were inserted into subcutaneous tissue of the patient's thigh, quantifying interstitial glucose concentration based on glucose oxidase reaction. Measurements were collected for up to 72h, while calibrations took place every eight hours, as recommended by the manufacturer. Arterial blood glucose (BG) values determined by blood gas analyzer Radiometer ABL800 were used as reference. Accuracy was illustrated in Clarke-error-grid and Bland-Altman-Plot. Non-parametric tests were performed (Mann-Whitney U Test and Spearman´s Correlation). Ethic vote Charité EA2/095/14.

## Results

**Figure 1:**
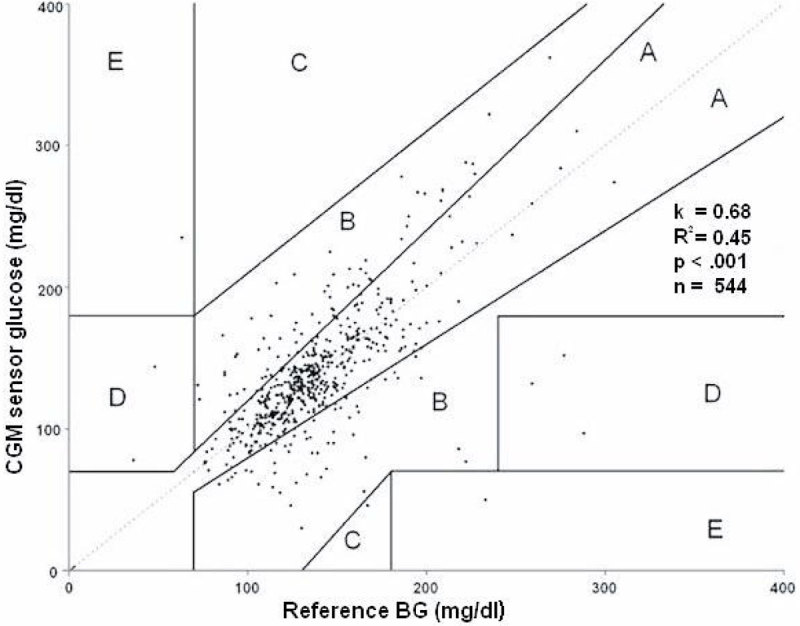
**Clarke-Error-Grid showing 544 values. Distribution: A = 76.7%, B = 21.9%, C--.2%, D = O.9%, E = O.4%.**

**Figure 2:**
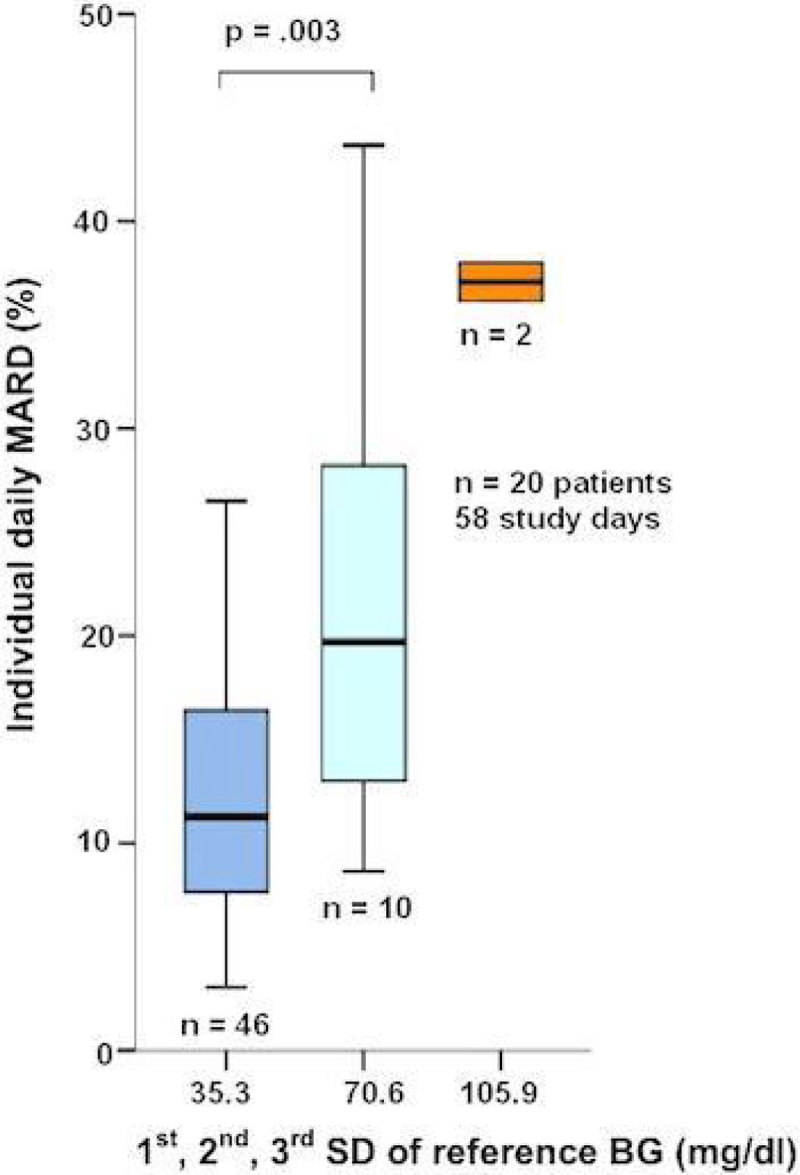
**Association MARD and individual BG variability shown in SD of reference BG on 58 study days. 1** ^**st**^ **SD (median = 11.3%), 2** ^**nd**^ **SD (median 19.7%).**

544 paired glucose values were generated from 32 sensors in 20 critically ill patients. Mean absolute relative difference (MARD) was 15.2% (95% CI 13.5%-17.0%). 60.7% of sensor data deviated ≤ 12.5% from reference BG (or were within ± 10mg/dl for readings < 100mg/dl), while 76.7% were within 20% of the reference. Clarke-error-grid is represented in Figure 1. In the Bland-Altman-Plot (graph not shown) mean bias was +1.55mg/dl and limits of agreement were +65.7mg/dl and -62.6 mg/dl (mean bias ± 1.96x standard deviation (SD)). We identified that the BG variability, analyzed in SD, is significantly associated with CGM accuracy (Figure 2). Confirming these finding, SD per patient was positively correlated with MARD per patient (k = 0.68, p = .001, n = 20, R^2^ = 0.345, graph not shown). Furthermore, MARD from the CGM devices was significantly higher when BG was >180mg/dl (p = .038) or < 80mg/dl (p < .001), as compared to the reference range of 80mg/dl -180mg/dl (graph not shown).

## Conclusions

In our patients, subcutaneous CGM accuracy does not fulfill criteria specified in the consensus recommendations of the expert meeting published in 2013 ([1]). Accuracy deteriorated in patients with high blood glucose variability, as well as in the hypo- and hyperglycemic range. Following our data, we cannot recommend a clinical use of the investigated device.

## Grant Acknowledgment

Preliminary study within ECCRN research award for clinical nutrion 2013.
